# Unexpected Findings in Diffuse ST-Segment Depression and aVR ST-Segment Elevation

**DOI:** 10.3390/diagnostics16091300

**Published:** 2026-04-27

**Authors:** Mohamed El Mallouli, Amina El Bakkali, Usama Azziz, Pierre-Emmanuel Massart, Georgiana Pintea Bentea

**Affiliations:** 1 Cardiology Department, CHU Brugmann, 1020 Brussels, Belgium; 2 Emergency Department, CHU Brugmann, 1020 Brussels, Belgium; 3 Cardiology Department, CHU Namur Sainte Elisabeth, 5000 Namur, Belgium

**Keywords:** diffuse ST-segment depression, electrocardiogram, gastric perforation

## Abstract

Electrocardiographic changes resembling myocardial ischemia are rare in gastrointestinal emergencies. In particular, gastric perforation has been reported in association with ST-segment elevation, but not with ST-segment depression mimicking non-ST-segment elevation myocardial infarction (NSTEMI). We report the case of a 60-year-old woman presenting with sudden-onset epigastric pain radiating to the chest. She remained hemodynamically stable throughout her emergency department stay. On admission, the ECG showed diffuse ST-segment depression with ST-segment elevation in aVR. High-sensitivity troponin and inflammatory markers were within normal limits. Coronary angiography revealed no significant coronary stenosis, and left ventriculography demonstrated preserved left ventricular systolic function. Abdominal computed tomography showed abundant pneumoperitoneum, diffuse anterior gastric wall thickening, and moderate intraperitoneal fluid, findings highly suggestive of gastric perforation. The patient underwent laparoscopic gastric repair and abdominal lavage, with an uneventful postoperative recovery. A repeat ECG 24 h after surgery showed complete resolution of the ST-segment abnormalities. To our knowledge, this is the first reported case of gastric perforation presenting with diffuse ST-segment depression and aVR ST-segment elevation. Awareness of this presentation helps to broaden the spectrum of diagnostic possibilities and to plan appropriate diagnostic–therapeutic procedures.

**Figure 1:**
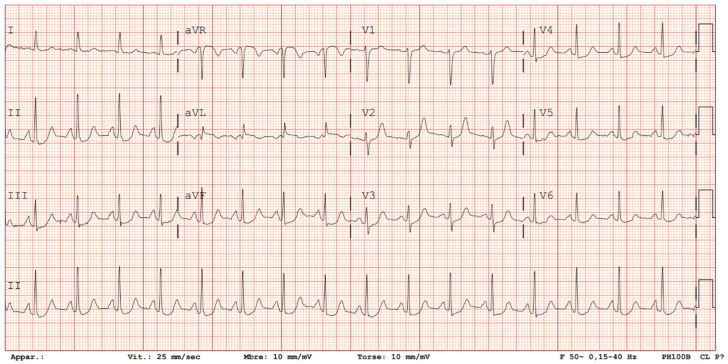
A 60-year-old woman presented to the emergency department with sudden-onset, severe epigastric pain, awakening her from sleep approximately 30 min prior to arrival. The pain was described as a tight, pressure-like sensation radiating to the chest with associated nausea (no vomiting). She had no previous similar episodes. Her medical history included untreated hypercholesterolemia, active tobacco use (one pack per day), and a paternal history of premature myocardial infarction. Upon arrival, she was alert and hemodynamically stable, with a blood pressure of 130/85 mmHg, heart rate of 100 bpm in sinus rhythm, temperature of 35 °C, and oxygen saturation of 97% on room air. Abdominal examination revealed marked epigastric tenderness with guarding and diffuse abdominal sensitivity to palpation. A 12-lead electrocardiogram (ECG) demonstrated diffuse ST-segment depression, most prominent in the inferior (II, III, aVF) and apical precordial (V3–V4) leads with relative sparing of leads I and aVL, accompanied by ST-segment elevation in lead aVR. Laboratory tests demonstrated normal high-sensitivity cardiac troponin (<3 ng/dL; normal <14 ng/dL) and C-reactive protein (1.7 mg/dL; normal <10 mg/dL), with only mildly elevated D-dimer levels at 606 ng/mL (normal <500 ng/mL).

**Figure 2:**
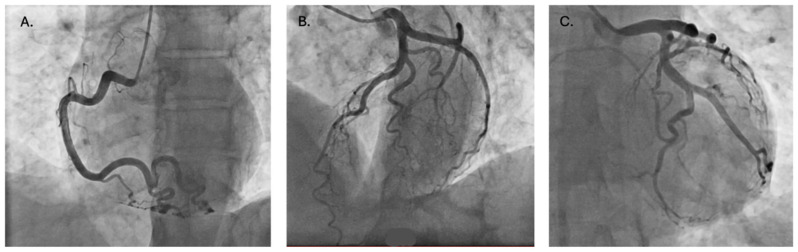
Due to persistent epigastric and chest discomfort despite adequate intravenous analgesia (paracetamol and morphine), and in the context of ECG findings, acute coronary syndrome could not be excluded, prompting urgent coronary angiography. This showed angiographically normal coronary arteries ((**A**)—right coronary artery; (**B**)—left anterior descending artery and diagonal branches; (**C**)—left main, circumflex, and obtuse marginal arteries) and preserved left ventricular systolic function without regional wall motion abnormalities on ventriculography Subsequent troponin levels, available post-procedure, remained within normal limits, confirming the absence of myocardial injury.

**Figure 3:**
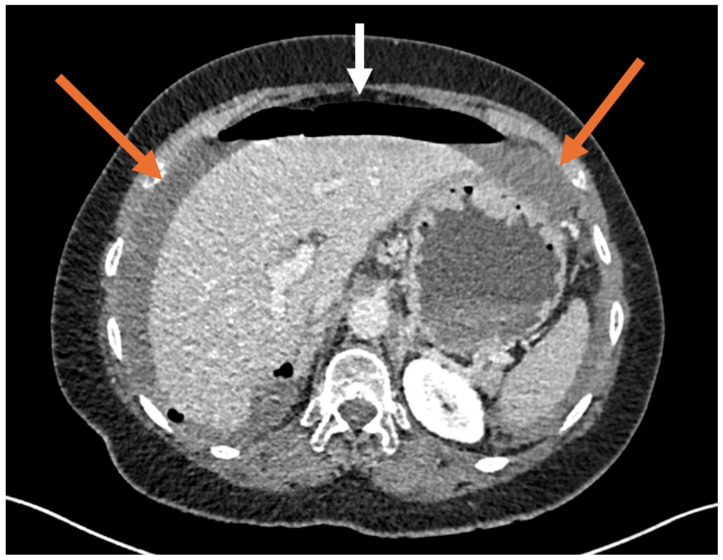
Given the persistence of epigastric tenderness and the absence of a cardiac etiology, an abdominal computed tomography scan was performed. It revealed diffuse pneumoperitoneum (white arrow), more prominent in the upper abdomen, along with diffuse thickening of the anterior gastric wall, thickened and infiltrated small bowel loops in the left flank and iliac fossa, and a moderate amount of intra-abdominal fluid (orange arrows), findings suggestive of a gastric perforation. The patient underwent emergency laparoscopic surgery, which confirmed a gastric perforation. A primary gastric suture repair (raffia) and peritoneal lavage were performed. Postoperative recovery was uneventful.

**Figure 4:**
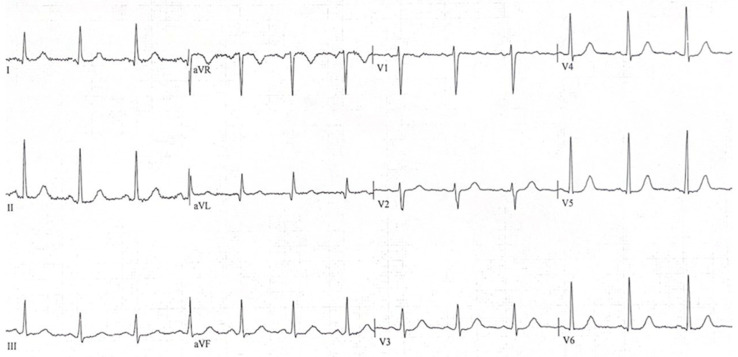
A repeat ECG performed 24 h after surgery showed complete normalization of the ST-segment abnormalities, consistent with a transient, non-ischemic, reversible electrocardiographic change secondary to the intra-abdominal process. Acute coronary syndromes may present with atypical symptoms, especially in women and elderly patients with cardiovascular risk factors. Gastrointestinal or abdominal pain is a well-recognized differential diagnosis of angina, particularly in inferior myocardial infarction [1]. Accordingly, obtaining a 12-lead ECG in patients with cardiovascular risk factors who present with epigastric discomfort is considered a prudent approach, as symptoms may represent an atypical manifestation of myocardial ischemia [2]. Our case represents the reverse scenario: a gastric perforation presenting with diffuse ST-segment depression and aVR ST-segment elevation. Early evidence suggested that this ECG pattern was highly specific for left main coronary ischemia or triple-vessel disease [3]. Consequently, guidelines—including the 2013 ACC/AHA STEMI guidelines—classified it as a ST-Segment Elevation Myocardial Infarction (STEMI) equivalent, warranting immediate coronary angiography [2]. However, more recent data indicate that diffuse ST-segment depression with ST elevation in aVR may also be seen in conditions such as sepsis, anemia, or aortic dissection [3]. A review of the literature identified only a few cases describing gastrointestinal perforation associated with ECG changes mimicking acute myocardial infarction [4,5,6,7,8]. In all reported instances, the presentation involved ST-segment elevation (STEMI-like pattern) with normal coronary angiography findings and resolution of ECG abnormalities after surgical management. Despite a comprehensive and structured literature search across the PubMed database, no additional case reports describing diffuse ST-segment depression and aVR ST-segment elevation in the setting of gastric perforation were identified. Several mechanisms have been proposed to explain ECG changes in non-cardiac conditions. Cardiac displacement due to pneumoperitoneum or gastric distension may modify the spatial orientation of the heart, thereby altering surface vector recordings [9]. Mechanical compression of the diaphragm or the cardiac base can transiently influence ventricular repolarization [10]. Additionally, viscero-cardiac autonomic reflexes may trigger transient electrophysiological disturbances or coronary vasospasm, producing pseudo-ischemic patterns [9,11]. Finally, systemic hypoperfusion or sepsis-related metabolic disturbances may contribute to transient repolarization abnormalities [3]. In nearly all reported cases, cardiac biomarkers remained normal or minimally elevated, coronary angiography showed no obstructive lesions, and ECG abnormalities resolved after correction of the underlying abdominal pathology [4,5,6,7,8]. These findings support the hypothesis of a functional extra-cardiac mechanism rather than true myocardial ischemia. Clinically, this case underscores the bidirectional diagnostic challenge between cardiac and abdominal pathologies. While ECG assessment is essential in patients presenting with symptoms suggestive of atypical acute coronary syndrome, clinicians should also be aware that acute abdominal emergencies can mimic cardiac ischemia. Persistent abdominal tenderness, normal cardiac biomarkers, and the absence of obstructive coronary disease should prompt further evaluation for an intra-abdominal cause. Importantly, performing coronary angiography in a patient with an unrecognized gastric perforation may be deleterious, as procedural anticoagulation can exacerbate intra-abdominal bleeding and complicate surgical management. This highlights the importance of a comprehensive, global clinical assessment. In this context, bedside echocardiography, awaiting serial troponin measurements, and careful consideration of epigastric guarding on physical examination might have helped steer the diagnostic process toward an abdominal etiology. On the other hand, this specific ECG pattern in the presence of chest-referred pain raises strong suspicion for acute coronary syndrome, is regarded in dedicated guidelines as a STEMI equivalent, and therefore warrants urgent cardiac catheterization given its potentially serious implications. Ideally, the chronology of complementary investigations should be optimized; nonetheless, both life-threatening cardiac and abdominal conditions must be excluded. In the presented case, coronary angiography was performed within approximately 30 min of the patient’s arrival to the emergency department, and abdominal CT imaging followed at around 1 h and 45 min. Ultimately, the diagnosis was not significantly delayed by this management approach. In conclusion, to our knowledge, this is the first reported case of gastric perforation presenting with diffuse ST-segment depression and aVR ST-segment elevation. Awareness of this presentation helps to broaden the spectrum of diagnostic possibilities and to plan appropriate diagnostic-therapeutic procedures.

## Data Availability

The data presented in this study are available on request from the corresponding author due to privacy reasons.

## Abbreviations

ECG	Electrocardiogram
NSTEMI	Non-ST-Segment Elevation Myocardial Infarction
STEMI	ST-Segment Elevation Myocardial Infarction

## References

[1] Canto J.G., Rogers W.J., Goldberg R.J., Peterson E.D., Wenger N.K., Vaccarino V., Kiefe C.I., Frederick P.D., Sopko G., Zheng Z.-J. (2012). Association of age and sex with myocardial infarction symptom presentation and in-hospital mortality. JAMA.

[2] Byrne R.A., Rossello X., Coughlan J.J., Barbato E., Berry C., Chieffo A., Claeys M.J., Dan G.-A., Dweck M.R., Galbraith M. (2023). 2023 ESC Guidelines for the management of acute coronary syndromes. Eur. Hear. Journal. Acute Cardiovasc. Care.

[3] Deng B., Liu W., Chu Q. (2026). The Prognostic-Diagnostic paradox of lead aVR: From ‘STEMI-Equivalent’ to ‘High-Risk NOMI’ in the era of precision cardiology. Int. J. Cardiol. Heart Vasc..

[4] Intan R.E., Hasibuan G., Gandi A., Alkaff F.F. (2021). Gastric perforation mimicking ST-segment elevation myocardial infarction. BMJ Case Rep..

[5] Vutthikraivit W. (2020). Perforated gastric ulcer with ST-segment elevation mimicking acute myocardial infarction. Southwest Respir Crit Care Chron..

[6] De Corcuera S.G., Sánchez-González J., Broncano Á., López-Vela J., Piñero P. (2023). Acute gastric perforation mimicking ST-segment elevation myocardial infarction. Rev. Esp. Cardiol. (Engl. Ed.).

[7] Hoang L., Labib S.B., Lentz C.W., Parker A., Motarjeme A. (2018). Gastric ulcer perforation into the pericardium presenting as ST-segment elevation myocardial infarction. Proc. Bayl. Univ. Med. Cent..

[8] Romero J., Shamoon Y., Santana M., Abboud R., Romero A.L., Reyes D., Pullatt R. (2024). Small bowel perforated viscus mimicking inferior wall ST-elevation myocardial infarction. JACC Case Rep..

[9] Zhang J., Basrawala H., Patel S., Girn H., Eyvazian V., Wang L., Ostrzega E. (2020). Gastrointestinal distention masquerading as ST-segment elevation myocardial infarction. JACC Case Rep..

[10] Khurana K.V., Ranjan A. (2022). ST-segment elevation in conditions of non-cardiovascular origin: A narrative review. J. Family Med. Prim. Care.

[11] Coppola G., Carità P., Corrado E., Borrelli A., Rotolo A., Guglielmo M., Nugara C., Ajello L., Santomauro M., Novo S. (2013). ST-segment elevation: Always a marker of acute myocardial infarction?. Int. J. Cardiol..

